# Salivary IL-6 as a Biomarker for Early Childhood and Rampant Caries: Insights from a Cross-Sectional Study

**DOI:** 10.3390/biomedicines13020293

**Published:** 2025-01-24

**Authors:** Vivek Padmanabhan, Md Sofiqul Islam, Kusai Baroudi, Nallan C. S. K. Chaitanya, Nada Tawfig Hashim, Manjunatha Goud, Muhammed Mustahsen Rahman, Dileep Sharma, Sheela Haridas, Mohamed Ahmed Abdel Baki, Rajaa Mhd Ghaleb Almasri

**Affiliations:** 1 RAK College of Dental Sciences, RAK Medical and Health Sciences University, Ras Al Khaimah P.O. Box 12973, United Arab Emirates; sofiqul.islam@rakmhsu.ac.ae (M.S.I.);; 2 Department of Clinical Sciences, College of Dentistry, Ajman University, Ajman P.O. Box 346, United Arab Emirates; d_kusai@yahoo.co.uk; 3 RAK College of Medical Sciences, RAK Medical and Health Sciences University, Ras Al Khaimah P.O. Box 11172, United Arab Emirates; 4 School of Health Sciences, The University of Newcastle, Callaghan, NSW 2308, Australia

**Keywords:** dental caries, saliva, interleukin-6, inflammation, children

## Abstract

**Objectives:** This study aimed to explore the levels of salivary interleukin-6 (IL-6) in children diagnosed with Early Childhood Caries (ECC) and Rampant Caries (RC). Additionally, it sought to determine whether salivary IL-6 levels vary with the increased activity or number of carious lesions in children with ECC and RC. **Methods:** A cross-sectional study was conducted at RAK College of Dental Sciences (RAKCODS), Ras Al Khaimah, UAE. Ethical approval was obtained, and 100 children aged 4 to 12 years were selected. Participants were divided into ECC and RC groups, each containing study and control subgroups of 25 children each. The study groups were children who had active carious lesions and the control groups were those children who had no active carious lesions. All of these children were screened and included as a part of the community engagement of the clinics. Saliva samples were collected and analyzed for IL-6 levels using an ELISA kit. Dental examinations and data collection adhered to standardized procedures, including specific clinical criteria for diagnosing ECC and RC and calibrated examiners to ensure consistent clinical assessments. Statistical analyses were conducted to compare salivary IL-6 levels between the study and control groups of each RC and ECC group and a test was also performed to assess the relationship between salivary IL-6 levels and the severity of dental caries. **Results:** This study found significantly higher mean salivary IL-6 levels in children with ECC (19.023 pg/mL) and RC (21.45 pg/mL) compared to their respective control groups (ECC: 6.42 pg/mL, RC: 11.43 pg/mL), with *p*-values < 0.0001. Strong positive correlations were observed between IL-6 levels and increased caries activity or severity, with Pearson’s correlation coefficients of 0.961 for ECC and 0.954 for RC. **Conclusions:** This study concludes that significantly elevated salivary IL-6 levels are observed in children with ECC and RC. Furthermore, salivary IL-6 levels were found to rise in correlation with the increase in number of carious lesions. These findings support the potential of salivary IL-6 as a biomarker for assessing caries severity and highlight the need for future research to explore diverse populations and additional clinical parameters.

## 1. Introduction

Dental caries is a biofilm-mediated, diet-modulated, multifactorial, non-communicable, dynamic disease that leads to net mineral loss in dental hard tissues [1]. Its development is influenced by biological, behavioral, psychosocial, and environmental factors, resulting in the formation of caries lesions [1,2]. Despite advances in dental care, caries remains a leading cause of tooth loss and morbidity, particularly in underserved populations [2]. Preventing dental caries is critical to reducing the burden of oral diseases, and it involves understanding its etiology, risk factors, and early detection methods [1,2,3]. Effective prevention and management strategies can mitigate the adverse effects of caries, improving health outcomes and reducing the healthcare costs and burden of the disease [1,3,4,5].

Dental caries can be classified into several types, including Early Childhood Caries (ECC) and Rampant Caries (RC) [4]. ECC, defined as the presence of one or more decayed, missing, or filled tooth surface in any primary tooth in children under six years of age, is a particularly aggressive form of caries [5]. It is associated with significant pain, infection, and difficulties in eating, speaking, and learning, severely affecting children’s development and well-being. RC is characterized by rapid and extensive decay involving multiple teeth, often affecting both primary and permanent teeth [6,7]. It typically occurs in older children and adolescents and can lead to severe oral health issues if not promptly addressed. Understanding the specific characteristics and progression of ECC and RC is crucial for developing targeted prevention and treatment approaches [8,9,10].

Saliva, a complex biofluid, plays a pivotal role in oral health by maintaining the integrity of oral tissues, facilitating digestion, and exerting antimicrobial effects [7]. Saliva plays a crucial role in maintaining oral health and preventing dental caries. It helps neutralize acids produced by bacteria in dental biofilms, provides essential minerals like calcium and phosphate for the remineralization of tooth enamel, and contains antimicrobial proteins that inhibit bacterial growth [7,8]. Additionally, saliva aids in the clearance of food particles and sugars from the mouth, reducing substrate availability for cariogenic bacteria. Therefore, adequate saliva production and composition are vital in mitigating the risk and progression of dental caries [7,8]. Recently, saliva has garnered attention as a valuable diagnostic tool due to its non-invasive collection, ease of sampling, and rich content of biomarkers reflecting both local and systemic health [9]. Salivary diagnostics offer a promising avenue for the early detection and monitoring of various diseases, including dental caries. Analyzing salivary components can provide insights into the pathophysiology of caries and identify individuals at higher risk, enabling timely intervention and personalized care [7,9,11,12,13]. Recent research highlights salivary biomarkers—including cytokines, enzymes, and nucleic acids—that can reliably reflect systemic inflammation and disease progression. These biomarkers enable early diagnosis, monitoring of treatment responses, and potentially the predicting of outcomes, all through simple and stress-free collection methods [14,15].

Interleukin-6 (IL-6) is a multifunctional cytokine involved in immune response, inflammation, and hematopoiesis [10]. It is produced by various cell types, including macrophages, fibroblasts, and endothelial cells, in response to infections and tissue injury. Elevated levels of IL-6 are associated with numerous inflammatory conditions and diseases, making it a key biomarker in medical diagnostics [12]. In the context of dental health, IL-6 has been implicated in the inflammatory response to cariogenic bacteria and the progression of caries [11,12]. Particularly in ECC and RC, where rapid tissue destruction occurs, IL-6 levels in saliva may reflect the underlying inflammatory processes and serve as an indicator of disease severity and activity [14]. This study aimed to investigate the relationship of salivary IL-6 levels in children with ECC and RC. Limited research has explored the correlation between salivary IL-6 and dental caries, particularly in the context of ECC and RC. This study was therefore designed to address this gap. The null hypothesis of this study posits that no relationship exists between salivary IL-6 levels and the presence of ECC or RC in children. The research aims to investigate whether a significant association exists between salivary IL-6 levels in children with and without ECC/RC. Furthermore, this study seeks to explore whether salivary IL-6 levels vary with increased caries activity, particularly as the number of carious lesions increases in children diagnosed with ECC and RC.

## 2. Materials and Methods

This cross-sectional study was conducted at RAK College of Dental Sciences (RAKCODS), part of RAK Medical and Health Sciences University (RAKMHSU) in Ras Al Khaimah, United Arab Emirates. The primary aim was to investigate the relationship between salivary IL-6 levels in children diagnosed with Early Childhood Caries (ECC) and Rampant Caries (RC) compared to those without active carious lesions. Additionally, this study explored whether salivary IL-6 levels varied with increasing severity or the number of dental caries. This study received ethical approval from the university’s Research and Ethics Committee and the RAK Research and Ethics Committee, Ministry of Health (Approval numbers: RAKMHSU-REC-015-2023/24-UG-D and MOHAP-REC-REF-24/37-2024-UG-D, Approval date: 13 October 2023). Saliva samples were collected from participants during visits to the Pediatric Dentistry clinics, and IL-6 levels were analyzed in the university’s biochemistry lab. Data collection occurred between November 2023 and May 2024.

### 2.1. Sample Size and Participant Selection

This study focused on children aged 4 to 12 years. Those younger than 5 years and 11 months were placed in the ECC group, while those aged 6 to 12 years were assigned to the RC group. At RAKCODS clinics, children are initially screened as part of community engagement, so both caries-free and caries-active children participate, simplifying recruitment. The pediatric dentistry clinic operates four evenings weekly, seeing an average of 3–4 new ECC patients and 4–5 new RC patients per week. Accordingly, this study aimed to enroll 60–70 children with ECC and 70–80 children with RC, including those with and without active caries. Using the Raosoft Online Sample Size Calculator with a 5% margin of error and a 90% confidence level, a sample size of 100 participants was deemed optimal. This included 25 children each in the study and control groups for ECC and RC. Participants were grouped based on caries status. Study groups comprised children with at least five active carious lesions (25 from ECC and 25 from RC), while control groups included children without active carious lesions (25 from ECC and 25 from RC). To ensure a representative sample, inclusion criteria required a minimum of five active carious lesions for the study groups. Children with medical conditions, medication histories, or hospitalization that could influence study variables, as well as those who did not provide consent, were excluded.

### 2.2. Study Protocol and Procedures

Children aged 4–12 years visiting RAKCODS pediatric clinics for dental treatment were invited to participate after obtaining parental consent. Group I (ECC) included children under 5 years and 11 months, while Group II (RC) included children aged 6–12 years. Participants and their parents were instructed to refrain from eating or drinking for at least two hours before saliva collection and were asked to rinse their mouths prior to the procedure. Saliva collection involved unstimulated saliva samples obtained using the Coachman’s Position [8], where participants bent their heads forward, relaxed, and passively let the saliva pool for 5 min, after which it was collected using Salimetrics SalivaBio^®^ Children’s Swab [8]. To minimize bias, co-investigators received comprehensive training in data recording and clinical procedures. A designated investigator recorded data during clinic visits, while another conducted intraoral examinations and saliva collection. Basic demographic information was recorded, and oral screening sheets used in community service initiatives documented caries status, including the DMFT/dmft index. This structured methodology ensured a consistent and reliable assessment of salivary IL-6 levels and their potential correlation with caries activity.

Samples were stored at 4 °C in an icebox and delivered to the laboratory within 20 min for analysis using an Enzyme-Linked Immunosorbent Assay (ELISA) kit [8] (Salimetrics High Sensitivity Human IL-6 ELISA Kit, Salimetrics LLC, Carlsbad, CA, USA) [8]. Saliva samples were collected, frozen at −20 °C within 4 h, then thawed, vortexed, and centrifuged (1500× *g*, 15 min). Following Salimetrics IL-6 ELISA kit guidelines, reagents were prepared, and samples diluted 5×. Standards, controls, and diluted samples (100 µL each) were added to wells, with two wells as zero standards. Plates were mixed at 500 rpm at room temperature, washed, then treated sequentially with a 1:500 diluted antibody conjugate, 1:100 diluted Streptavidin-HRP, TMB substrate, and stop solution before reading at 450 nm [8].

### 2.3. Statistical Analysis

Data were analyzed using Statistical Package for the Social Sciences (SPSS, version 29.0, IBM Corp., Armonk, NY, USA). The normality assumption for salivary IL-6 levels in the study and control groups, within both ECC and RC categories, was assessed using QQ plots and measures of skewness and kurtosis. Since the data exhibited a skewed distribution with outliers, the Wilcoxon signed-rank test was used to compare salivary IL-6 levels between age-matched study and control groups. The influence of severity of dental caries on salivary IL-6 levels in ECC and RC categories was evaluated using the Pearson Correlation Coefficient. A *p*-value less than 0.05 was considered statistically significant.

## 3. Results

Children who participated in this study belonged to the age group of 4–12 years. The mean age of the ECC group was 4.64 ± 0.44 and that of RC group was 9.39 ± 1.85 years. Table 1 provides a baseline comparison between the study and control groups of each ECC and RC group. Both the ECC and RC cohorts demonstrate a high level of baseline equivalence between the control and study groups, with just a slight, non-significant trend in age noted within the RC group. This comparison of the baseline data indicates that any future differences in outcomes are probably not a result of baseline variations but rather linked to the interventions or exposures under investigation. Children with any comorbidities were not included in the current research within either the study or control groups.

**Figure 1:**
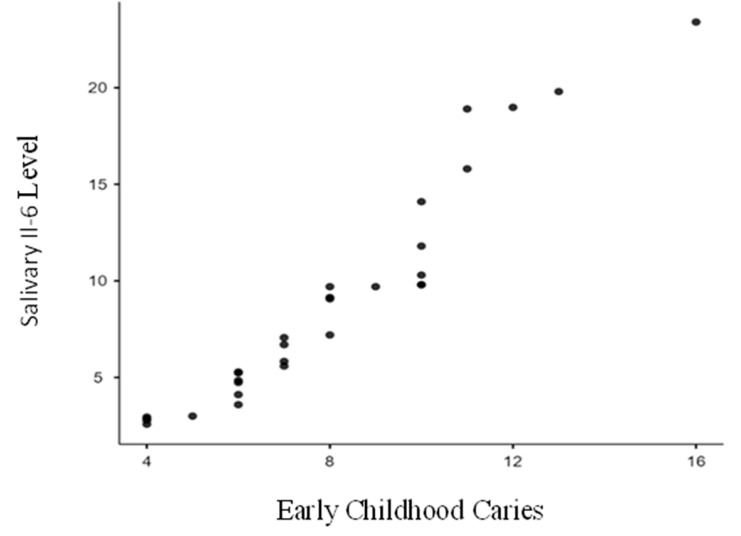
Correlation between severity of Early Childhood Caries and salivary interleukin-6 levels (salivary IL-6 levels). As Early Childhood Caries (dmft/DMFT) increases, the salivary IL-6 levels also increase. There is a strong positive correlation (0.961) which is statistically significant (*p* < 0.0001).

**Figure 2:**
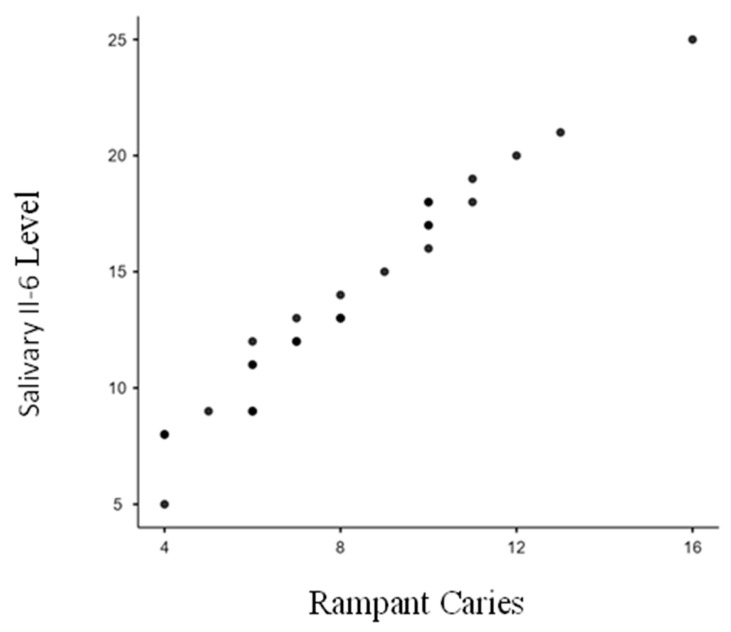
Correlation between Rampant Caries (DMFT) and salivary interleukin-6 levels (salivary IL-6 levels). As Rampant Caries (dmft/DMFT) increases, the salivary IL-6 levels also increase. There is a strong positive correlation (0.954) which is statistically significant (*p* < 0.0001).

Table 2 showcases the findings for Group I (ECC), highlighting the comparison of median salivary IL-6 levels between children with active carious lesions (study group) and those without (control group). The study group exhibited median levels of 20.7 pg/mL, significantly higher than the control group’s 5.6 pg/mL, with a *p*-value less than 0.0001 (*p* < 0.0001). A Pearson’s Correlation Coefficient of 0.961 (*p* < 0.0001) demonstrated a strong positive correlation between salivary IL-6 levels and ECC severity (Figure 1). Similarly, Table 3 presents result for Group II (RC), comparing median salivary IL-6 levels between the study and control groups. The study group’s median level was 21.0 pg/mL, significantly greater than the control group’s 14.2 pg/mL, with a *p*-value below 0.0001 (*p* < 0.0001). The Pearson’s Correlation Coefficient of 0.954 (*p* < 0.0001) indicated a strong positive correlation between salivary IL-6 levels and RC severity (Figure 2). Overall, this study revealed a significant relationship between ECC, RC, and elevated salivary IL-6 levels, with higher IL-6 levels strongly associated with the increased severity of both ECC and RC. This study demonstrates a statistically significant relationship between the presence of ECC, RC, and salivary IL-6 levels when comparing the study and control groups. Additionally, it reveals a positive correlation between salivary IL-6 levels and the increasing severity of ECC and RC within the study groups.

The median salivary IL-6 levels of the study group (21 pg/mL) are significantly higher than those of the control group (14.2 pg/mL) and the results are statistically significant (*p*-value < 0.0001).

The median salivary IL-6 levels of the study group (20.7 pg/mL) are significantly higher than those of the control group (5.6 pg/mL) and the results are statistically significant (*p*-value < 0.0001).

## 4. Discussion

The primary aim of this study was to explore the connection between Early Childhood Caries (ECC), Rampant Caries (RC), and salivary IL-6 levels. Additionally, the research sought to determine if salivary IL-6 levels rise in conjunction with the severity of or increase in the number of dental carious lesions, specifically assessing whether an increased number of active carious lesions correlates with higher salivary IL-6 levels. This investigation aimed to delve into the intricate interactions among these critical variables to uncover insights that could guide the creation of improved oral health strategies and interventions for children.

Salivary IL-6 is a cytokine, an important signaling molecule in the immune system, which plays a key role in inflammation and the body’s response to infections [16,17]. Cariogenic bacteria, such as *Streptococcus mutans* and *Lactobacillus*, contribute to the development of dental caries by producing acids that lead to enamel demineralization [1,2,3]. The presence of these bacteria can trigger an inflammatory response in the oral mucosa, resulting in elevated IL-6 levels in saliva [1,2]. Therefore, higher salivary IL-6 levels may be associated with increased bacterial load and activity, reflecting the body’s inflammatory response to cariogenic microorganisms [12,17,18]. Monitoring salivary IL-6 levels can therefore serve as a useful biomarker for diagnosing and assessing the severity of oral diseases, enabling more targeted and effective treatment strategies to maintain and improve oral health [12,18,19].

In children with Early Childhood Caries (ECC) under the age of 5 years and 11 months, the current study identifies significantly elevated salivary IL-6 levels, aligning with findings from studies worldwide where it was also seen that there is an increased salivary IL-6 activity with the presence of dental caries [15,16,17,18]. The chronic inflammatory response observed in ECC is attributed to the continuous presence of cariogenic bacteria, such as *Streptococcus mutans*, which initiate and perpetuate immune reactions [20]. The release of IL-6, a critical cytokine in inflammation and infection control, is part of the body’s defense mechanism against bacterial invasion and subsequent tissue damage [15,16,17,18,20,21]. Studies have shown a strong correlation between ECC severity and increased salivary IL-6 levels [20,21]. Studies have also reported persistently elevated IL-6 levels in children with chronic carious lesions, reinforcing its role as an inflammatory marker and highlighting its association with ECC progression, where higher bacterial loads correspond to increased cytokine release [17,21]. These findings could be suggestive of the idea that elevated IL-6 levels are a reflection of the body’s ongoing attempt to manage inflammation and control infection. Moreover, poor gingival health, often associated with ECC, contributes to the exacerbation of inflammation. Gingival tissue breakdown not only facilitates bacterial colonization but also impedes effective plaque control, thus perpetuating the cycle of caries and inflammation [17]. The persistent inflammatory response in ECC underscores the need for preventive measures such as improved oral hygiene, fluoride application, and dietary modifications. Similarly, in children between the ages of 6 and 12 years with Rampant Caries (RC), salivary IL-6 levels were significantly elevated, indicative of a heightened inflammatory response. RC is characterized by rapid and widespread decay, affecting multiple teeth, often due to poor oral hygiene, excessive sugar intake, and insufficient fluoride exposure [7,8,20]. The extensive bacterial activity associated with RC leads to severe tissue destruction, triggering robust immune responses and increased IL-6 production [20]. Research corroborates these findings, with studies conducted elsewhere which highlight the association between RC severity and elevated inflammatory markers, including IL-6 [18,19,20,21]. The widespread infection in RC cases plausibly necessitates an intense immune reaction, as reflected in the high salivary IL-6 levels observed in affected children. Additionally, studies suggest that these elevated cytokine levels not only mark disease severity but also indicate systemic stress caused by unresolved infection and inflammation [18,19,20,21,22,23,24].

The current research revealed increased levels of salivary interleukin-6 (IL-6) in children suffering from Early Childhood Caries (ECC) and Rampant Caries, indicating a notable connection between proinflammatory cytokines and the severity of dental decay. Previous studies have shown that salivary IL-6 levels decrease with improved oral hygiene [25], supporting earlier findings that cytokines like tumor necrosis factor-alpha (TNF-α), IL-6, and interleukin-8 (IL-8) are closely associated with the progression of dental caries [26]. Furthermore, investigations involving mother–child pairs have demonstrated that elevated levels of these cytokines correlate with caries severity, with contributing factors such as obesity and high sugar intake significantly influencing this relationship [27]. The findings of the present research reinforce the idea that both biological indicators and lifestyle choices play a role in the development of dental caries, implying that improving dietary practices and addressing obesity may be essential strategies for mitigating inflammatory responses and enhancing oral health in affected children.

Our findings align with global research, revealing a strong association between IL-6 levels and the severity and extent of carious lesions, suggesting that reducing IL-6 may alleviate inflammation related to dental caries [21,22,23,24,25]. However, factors like nutritional status, access to dental care, and public health policies can influence inflammatory profiles and potentially account for variations in the results. Therefore, a comprehensive approach that considers genetic, environmental, and cultural factors is essential for accurate interpretation. Future studies should include larger populations and a wider range of clinical and biochemical measures to better understand the role of salivary IL-6 in dental caries and to develop more targeted oral health strategies. Notably, this research is pioneering in evaluating salivary IL-6 levels in both Early Childhood Caries (ECC) and Rampant Caries (RC), conditions primarily affecting the pediatric population.

The relatively small sample size of the current study limits the generalizability of the findings, as a larger cohort could provide more robust statistical power and a clearer understanding of the observed associations. Moreover, potentially confounding factors such as varying dietary habits, differences in oral hygiene practices, and fluctuating stress levels were not extensively controlled. These factors could influence salivary IL-6 levels, potentially skewing the results. Future research should involve larger and more diverse populations with meticulous control of these variables, while exploring a broader range of biochemical and clinical parameters. It is also important to investigate possible differences in salivary IL-6 levels between males and females, as gender-specific factors may affect immune responses. Longitudinal studies could further clarify the causal role of salivary IL-6 in caries progression, ultimately supporting the development of more effective oral health strategies for children.

## 5. Conclusions

In conclusion, the current study indicates that salivary IL-6 levels increase in children with ECC and RC. This study also found that the levels of salivary IL-6 increase with increases in the severity and number of dental caries activity in these children. Future research should build on these findings by incorporating diverse populations and a broader range of variables to refine oral health interventions and enhance our understanding of IL-6’s role in dental caries.

## 6. Recommendations

Based on these conclusions, general and pediatric dentists should consider incorporating regular salivary IL-6 assessments as part of their diagnostic and monitoring protocols for children with dental caries. By identifying elevated IL-6 levels, dentists can more accurately gauge the severity of carious lesions and tailor treatment plans accordingly. Additionally, enhancing patient education on the importance of oral hygiene, nutrition, and timely dental care can help manage and prevent the progression of carious conditions. Collaboration with researchers and public health professionals to stay informed about advances in biomarkers and treatment strategies will further support effective, evidence-based care for pediatric patients.

## Figures and Tables

**Table 1:** Comparison of baseline data of ECC and RC groups.

Characteristics	ECC Control	ECC Study	*p* Value (ECC)	RC Control	RC Study	*p* Value (RC)
**Age**	4.65 ± 0.43	4.64 ± 0.44	1.000	9.56 ± 2.28	9.23 ± 1.43	0.080
**Gender (M/F)**	12/13	13/12	0.705	13/12	13/12	1.000
**Height (m)**	1.08 ± 0.02	1.07 ± 0.02	1.000	1.34 ± 0.03	1.33 ± 0.03	0.534
**Weight (kg)**	15.84 ± 1.85	15.96 ± 1.87	1.000	27.14 ± 1.66	27.14 ± 1.66	1.000
**BMI (kg/m²)**	13.59 ± 1.67	13.62 ± 1.90	0.948	15.23 ± 1.12	15.34 ± 1.20	0.707

**Table 2:** Comparison of median salivary interleukin-6 levels (salivary IL-6 levels) between study and control groups of children with Early Childhood Caries.

Variable	Group	Median	IQR	*p* Value
**Age**	Control	4.67	0.58	
	Study	4.67	0.58	
**IL-6 (pg/mL)**	Control	5.6	0.839	**<0.0001**
	Study	20.7	4.015	

**Table 3:** Comparison of median salivary interleukin-6 levels (salivary IL-6 levels) between study and control groups of children with Rampant Caries.

Variable	Group	Median	IQR	*p* Value
**Age**	Control	8.92	4.09	
	Study	8.17	1.92	
**IL-6 (pg/mL)**	Control	14.2	2.681	**<0.0001**
	Study	21	4.916	

## Data Availability

The original contributions presented in this study are included in the article. Further inquiries can be directed to the corresponding authors.
